# Effect of poly (lactic-co-glycolic acid) polymer nanoparticles loaded with vancomycin against *Staphylococcus aureus* biofilm

**DOI:** 10.1186/s12896-023-00811-8

**Published:** 2023-09-18

**Authors:** Ellahe Nouruzi, Seyed Mostafa Hosseini, Babak Asghari, Reza Mahjoub, Ehsan Nazarzadeh Zare, Mohammad-Ali Shahbazi, Fereshte Kalhori, Mohammad Reza Arabestani

**Affiliations:** 1 Department of Microbiology, Faculty of Medicine, Hamadan University of Medical Sciences, Hamadan, IR Iran; 2 Infectious Disease Research Center, Hamadan University of Medical Sciences, Hamadan, IR Iran; 3 Department of Pharmacology and Toxicology, School of Pharmacy, Medicinal Plants and Natural Products Research Center, Hamadan University of Medical Sciences, Hamadan, Iran; 4 School of Chemistry, Damghan University, Damghan, IR Iran; 5 Department of Biomedical Engineering, University Medical Center Groningen, University of Groningen, Antonius Deusinglaan 1, 9713 AV Groningen, The Netherlands; 6 Biotechnology department, Hamadan University of Medical Sciences, Hamadan, IR Iran

**Keywords:** *Staphylococcus aureus*, Vancomycin, PLGA, Lysostaphin, Biofilm

## Abstract

*Staphylococcus aureus* is a unique challenge for the healthcare system because it can form biofilms, is resistant to the host's immune system, and is resistant to numerous antimicrobial therapies. The aim of this study was to investigate the effect of poly (lactic-co-glycolic acid) (PLGA) polymer nanoparticles loaded with vancomycin and conjugated with lysostaphin (PLGA-VAN-LYS) on inhibiting *S. aureus* biofilm formation. Nano drug carriers were produced using the double emulsion evaporation process. we examined the physicochemical characteristics of the nanoparticles, including particle size, polydispersity index (PDI), zeta potential, drug loading (DL), entrapment efficiency (EE), Lysostaphin conjugation efficiency (LCE), and shape. The effect of the nano drug carriers on *S. aureus* strains was evaluated by determining the minimum inhibitory concentration (MIC), conducting biofilm formation inhibition studies, and performing agar well diffusion tests. The average size, PDI, zeta potential, DL, EE, and LCE of PLGA-VAN-LYS were 320.5 ± 35 nm, 0.270 ± 0.012, -19.5 ± 1.3 mV, 16.75 ± 2.5%, 94.62 ± 2.6%, and 37% respectively. Both the agar well diffusion and MIC tests did not show a distinction between vancomycin and the nano drug carriers after 72 h. However, the results of the biofilm analysis demonstrated that the nano drug carrier had a stronger inhibitory effect on biofilm formation compared to the free drug. The use of this technology for treating hospital infections caused by the *Staphylococcus* bacteria may have favorable effects on staphylococcal infections, considering the efficacy of the nano medicine carrier developed in this study.

## Introduction

*Staphylococcus aureus* is a gram-positive opportunistic bacteria that frequently causes skin and soft tissue infections [1, 2]. This bacterium's primary natural habitat is the surface of human skin and mucous membranes. This bacteria is one of the main causes of hospital- and community-acquired infections, and it puts a significant strain on the healthcare system [1, 2]. *S. aureus* colonization occurs in two ways: intermittent carriers (75–80%) and persistent carriers (20–25%). *S. aureus* nasal carriage is directly correlated with the incidence of hospital-acquired infections [3]. *S. aureus* is one of the typical causes of infections related to medical equipment, skin and soft tissue infections, infected endocarditis, and osteomyelitis [4]. Multidrug-resistant strains of *Staphylococcus aureus*, especially methicillin resistant (MRSA) strains, pose a significant threat to human health, leading to severe morbidity and mortality. These strains are particularly problematic in healthcare settings, such as hospitals, as well as among otherwise healthy individuals. MRSA strains demonstrate resistance to multiple antimicrobials, including penicillins, carbapenems, and cephalosporins. This resistance is required through the acquisition of the mobile staphylococcal cassette chromosome mec (SCC mec), which carries the mecA gene. The mecA gene encodes for altered penicillin-binding proteins (PBP2a) consequently rendering their treatment challenging [5]. Glycopeptide antibiotics, particularly vancomycin, have consistently been the preferred therapy for treating MRSA infections [6]. Vancomycin has traditionally been regarded as the ultimate treatment option for MRSA infections. Nevertheless, its extensive utilization has led to the emergence of various strains, including vancomycin-intermediate *Staphylococcus aureus* (VISA) and vancomycin resistant *Staphylococcus aureus* (VRSA) [5]. The side effects of vancomycin include hypotension, nephrotoxicity, ototoxicity, tachycardia, chills, and fever [7]. Cell-to-cell adhesion takes place once the bacterium adheres to either a living surface or a non-living surface. Subsequently, the bacteria undergo multiplication, resulting in the formation of a biofilm consisting of multiple layers. Within this biofilm, a cluster of bacteria is enclosed by an extracellular polymeric matrix, which is produced by the bacteria themselves [3, 8, 9]. The hydrated extracellular polymeric matrix is primarily composed of polysaccharides, extra cellular DNA (eDNA), and proteins. This matrix serves as a protective shield for the bacteria, safeguarding them against heat, radiation, the immune system, and antibiotic treatment [3, 8, 10]. It is challenging to eliminate these infections due to the development of biofilm and cell encapsulation in the polymer matrix, which decreases sensitivity to antimicrobial drugs and host defenses [8]. Numerous treatment failures in clinical settings have been attributed to the fact that bacteria in biofilm form are 100–1000 times more resistant to antibiotics compared to planktonic bacteria [11]. As a result, biofilm-related infections are frequently chronic, recurring, and challenging to cure [11]. To solve these challenges, novel anti-biofilm therapies are required [12]. One of the most promising approaches currently being explored is the utilization of nanoparticles for drug delivery [11, 13]. Several studies have demonstrated that nanoparticles can effectively target biofilms and serve as potent weapon against biofilm-associated infections [13, 14]. Nanoparticles enhance the penetration of drugs into the deep layers of the biofilm. They enhance the bioavailability of antibiotics and the efficacy of the treatment [15–17]. The limitations of traditional therapy with vancomycin and the challenges in treating biofilm-related infections have led to the development of drug delivery systems based on nanoparticles: 1) It has been demonstrated that the sustained and controlled release of vancomycin from nanoparticles leads to the preservation of high levels of antimicrobial substance within the biofilm and enhances the drug’s interaction with bacterial cells. This increases the duration of antibiotic contact with bacteria within the biofilm and the antibacterial efficacy compared to free drug. 2) It improves the drug’s pharmacokinetics. 3) It reduces toxicity. 4) Nanoparticles, due to their size and surface properties, can penetrate the biofilm and consequently increase the local drug concentration in the deeper layers of the biofilm. 5) Nanoparticles can be targeted with surface ligands, and the drug can be released in a location near the biofilm. 6) Free drug may be susceptible to enzymatic and chemical degradation, whereas nanoparticles protect the drug from degradation and inactivation within the biofilm [18, 19]. Polylactic-co- glycolic acid (PLGA), one of several nanoparticles, has garnered particular interest because of its distinctive features. The FDA has approved PLGA, a biodegradable polymer with great biocompatibility, for delivering medications in a steady and controlled manner while guarding them from chemical and enzymatic destruction [16, 20–23]. Numerous studies have been conducted on the effectiveness of PLGA nanoparticles on bacterial biofilms and MRSA infections. A study by Anjum et al. [24] in 2019 in Malaysia demonstrated the effectiveness of PLGA nanoparticles loaded with xylitol on bacterial biofilms. These nanoparticles successfully penetrated the biofilm matrix [24]. The use of bacteriocins to treat biofilm infections has also been successful [25]. Methicillin-resistant *S. aureus* (MRSA) infections can be treated with lysostaphin enzyme, a bacteriocin that is beneficial alone or in conjunction with antibiotics for treating a variety of *Staphylococcus* infections in humans. Lysostaphin, a 27 KD metalloenzyme, is capable of precisely dissolving pentaglycine cross-bridges found in *S. aureus* cell walls. This enzyme kills planktonic *Staphylococcus aureus* strains and prevents the growth of bacteria in the biofilm [26–28]. Therefore, considering biofilm resistance and the aforementioned issues, the goal of this work was to create a novel technique to increase the efficiency of current antibiotics in preventing the formation of biofilms in *S. aureus* strains. As a result, PLGA-VAN-LYS nanoparticles were created using the double emulsion evaporation method, and their ability to prevent *S. aureus* biofilm formation was tested.

## Materials and methods

### Research materials

Vancomycin, polyvinyl alcohol (PVA), poly (lactic-co-glycolic acid) (PLGA) 50:50, and lysostaphin were all given by Sigma Aldrich (MO, USA). Sigma Aldrich (MO, USA) provided the 2-morpholinoethanesulfonic acid (MES), 1-ethyl-3-(3-dimethylaminopropyl) carbodiimide hydrochloride (EDC), and N-hydroxysulfosuccinimide (NHS). Both the MTT and Bradford protein test kits came from Kiazist in Iran. Merck (Darmstadt, Germany) furnished the blood agar, Mueller–Hinton broth, Mueller–Hinton agar, chloroform, and dichloromethane. Fetal bovine serum (FBS), penicillin, and streptomycin were bought from Gibco in the USA. The Dulbecco's Modified Egle's Medium (DMEM) was also used.

### Synthesis of PLGA-VAN

The double emulsion-solvent evaporation approach was used to create PLGA-VAN. In summary, 15 mL of chloroform was employed to dissolve 120 mg of PLGA polymer, which was then combined at 25 °C with magnetic stirring at 150 rpm for three hours. After that, the first emulsion (W1/O) was created by adding 24 mg of vancomycin to the PLGA-chloroform mixture. The primary emulsion was combined with 2% PVA and homogenized using an ultrasonic instrument (Bandelin Sopopuls, Berlin, Germany) at 45% amplitude (20 W) for one minute with a regular pulse rhythm (10 s on and 5 s off) to generate the secondary emulsion (W1/O/W2). Dropwise additions of the second emulsion were made into 20 mL of cold distilled water (4°C) with magnetic stirring for 30 min. Finally, PLGA-VAN was centrifuged (Eppendorf North America Co, US) at high speed for 20 min at 4 °C after being washed three times with sterile distilled water. To make the samples suitable for biological studies involving bacteria and cell lines, they underwent lyophilization at -80 ˚C using a vacuum pump (Christ, China) equipped with a condenser flow. Subsequently, the lyophilized nanoparticles were reconstituted in a solution and sterilized using 450 nm filters [29, 30].

### Lysostaphin conjugation

In accordance with a specific methodology, 10 mL of MES buffer (pH 5.0) were used to scatter 20 mg of lyophilized PLGA-VAN NPs. 1 mL of 0.1 M EDC and 1 mL of 0.7 M NHS, both dissolved in MES buffer with a pH of 5.0, were added to the NPs suspension to complete the activation process. The suspension was then gently stirred at room temperature for an hour. After that, the activated NPs were centrifuged (Eppendorf North America Co, US) (21,000 × g, 10 min, 4°C) to remove any residual reagents. The resulting pellets were redispersed in phosphate-buffered saline (PBS). To the suspensions, 3 mg of lysostaphin was added, and the mixture was homogenized using a vortex mixer. It was then incubated for 24 h at 4°C. A second centrifugation (21,000 × g, 10 min, 4˚C) was performed to get remove any unconjugated lysostaphin. The resulting pellets were resuspended in PBS. The effectiveness of lysostaphin conjugation to PLGA-VAN NPs was assessed using the Coomassie PlusTM (Bradford) test kit for the Bradford protein assay. Bovine serum albumin standards (0–50 mg/mL) were prepared by diluting them in PBS following the instructions provided with the Bradford kit (Kiazist, Iran). In the next step, the wells of an ELISA plate were filled with 180 µL of Bradford's reagent. Then, Bradford's reagent was added to each well, and 20 µL of the diluted standard solutions were pipetted into each well. The plate was incubated at room temperature for 5 min, and the optical density (OD) was assessed at wavelength of 595 nm [31].

### Characteristics of NPs

The Zetasizer Nano ZS 3600 equipment (Malvern Devices, Worcestershire, United Kingdom) was used to determine particle size, PDI, and Zeta potential after lyophilization using the Dynamic light Scattering (DLS) method [32].

### Morphology

Field emission scanning electronic microscopy, also known as a FE-SEM, was used to examine the morphological characteristics of NPs. In summary, 10 mg of lyophilized PLGA-VAN NPs were dissolved in 1 mL of distilled water before 2 µL of this suspension was applied to a glass surface. The suspension was dried and covered with a thin gold layer to prevent electrostatic charge during inquiry and analysis using FE-SEM (TSCAN, Czech Republic) [33].

### Determination of entrapment efficiency (EE %) and drug loading (DL %)

In accordance with recommendations made in the literature, an indirect approach (spectrophotometer) was used to determine how much vancomycin was loaded and encapsulated inside the synthesized NPs. First, 1 mL of distilled water was mixed with 5 mg of lyophilized nanoparticles before being vortexed. The next stage was centrifugation (37,000 × g for 20 min at 4 °C). Using a spectrophotometer (2100UV, USA) at a wavelength of 283 nm, the supernatant was examined. A standard curve was used to determine the drug's concentration. The following equations were applied in order to determine the amount of loaded and encapsulated drug [34]:

$$\mathrm{Entrapment}\;\mathrm{Efficiency}\;\mathrm{EE}\;\left(\%\right)=\frac{\mathrm{initial}\;\mathrm{drug}\;\mathrm{amount}-\mathrm{free}\;\mathrm{drug}\;\mathrm{amount}}{\mathrm{initial}\;\mathrm{drug}\;\mathrm{amount}}\times100$$

$$\mathrm{Drug}\;\mathrm{Loading}\;\mathrm{DL}\;(\%)=\frac{\mathrm{initial}\;\mathrm{drug}\;\mathrm{amount}-\mathrm{free}\;\mathrm{drug}\;\mathrm{amount}}{\mathrm{initial}\;\mathrm{PLGA}\;\mathrm{amount}}\times100$$

### Determination of stability of NPs

Regular time intervals were used to evaluate the stability of NPs. Using nano Zetasizer device (Malvern Devices, Worcestershire, United Kingdom), the particle size, zeta potential, and PDI were assessed at intervals of 0, 2, 4, 6, 8, and 12 months following lyophilization. At the same time, a spectrophotometer was used to calculate how much vancomycin was injected into the nanoparticles [30].

### Determination of drug release

The lyophilized nanoparticles (NPs) were precisely weighed and dissolved in 1 ml of release medium, put into a dialysis bag (cut-off 12,000, Dialysis tubing, Sigma Chem. Co., Missouri, USA), and put into a 40 mL release medium (PBS buffer, pH: 7.4) while being agitated magnetically at 100 rpm at 37 °C. At certain intervals, a spectrophotometer (2100UV, USA) was used to check the vancomycin content of 1 mL of the medium. To compare the outcomes of this series of tests with those of free vancomycin, the same technique was used with free vancomycin that had been inserted into dialysis bags and in the same medium. The medium was then sampled repeatedly throughout the same time periods, and the outcomes were examined. It should be emphasized that a brand-new, fresh medium in the exact same quantity was provided following each medium sampling [29].

### Fourier-transform infrared spectroscopy (FTIR) and differential scanning calorimetry (DSC)

To ascertain any potential interactions between vancomycin and the ingredients in PLGA-VAN NPs, a series of studies were conducted. This option was examined using FTIR analysis in the 400–4000 °C temperature range. At a rate of 10 °C/min, a DSC analysis was conducted from 20 to 400 °C [32].

### MTT assay

Using the MTT assay kit (Kiazist, Iran), the cytotoxicity assay test was conducted. A murine fibroblast cell line was used to test the cytotoxic effects of PLGA-VAN, Free vancomycin, lysostaphin, and pure PLGA. A 96-well clean plate, two channel reservoirs, solvent, and MTT reagent were all included in the kit. This stage involved counting 10^4^ L929 fibroblasts using the trypan blue staining method, transferring them to 96-well cell culture plates filled with Dulbecco's Modified Egle's Medium (DMEM) culture containing 10% FBS (fetal bovine serum) and 1% penicillin–streptomycin, and incubating them there for an overnight period of time at 37 °C with 5% CO_2_. After removing the DMEM medium, PLGA-VAN, Free vancomycin, lysostaphin, and pure PLGA were supplied together with DMEM containing 10% FBS and incubated for 24 h. Positive controls included wells with medium but no drug. Three times each were done for every experiment. Following a PBS wash to remove any remaining medications or polymers, each well received 150 µL of new DMEM devoid of fetal bovine serum. Next, 20 µL of the MTT test reagent were applied to each well. The plate was incubated at 37 °C with 5% CO_2_ for 3–4 h. After that, each well received a 100 µL solubilizer and an orbital shaker for 15 min in order to resolve formazan particles. In order to measure the absorbance at 570 nm, a 96-well ELISA plate reader was used. The percentage of cells in the positive control group that were absorbed (100 percent alive) was utilized to determine the cells' vitality [35].

### Bacterial strains

The bacterial strains used in this study are from Hamadan University of Medical Science in Hamadan, Iran. They are methicillin-sensitive *Staphylococcus aureus* (MSSA) (ATCC 25923), methicillin-resistant *Staphylococcus aureus* (MRSA) (ATCC 33591), and vancomycin intermediate resistant *Staphylococcus aureus* (VISA). Additionally, a clinical strain that was obtained and employed in this investigation originated from the Besat Hospital in Hamadan, Iran.

### Agar well diffusion and minimum inhibitory concentration (MIC)

Agar well diffusion and minimum inhibitory concentrations (MIC) testing were carried out in accordance with CLSI recommendations. Each well received 100 µL of various doses of PLGA-VAN, PLGA-VAN-LYS, and free vancomycin (5, 25, and 50 µg/mL) and was then incubated at 37 °C for 24, 48, and 72 h. The diameter of the inhibitory zone was then determined for each well. Sterilized 96-well cell culture plates were used for the MIC test. The PLGA-VAN, PLGA-VAN-LYS, and free vancomycin were delivered in a range of concentrations, and 100 µL of each concentration was applied to each microplate well aside from the positive control wells. 100 µL of Muller Hinton broth medium were then given to each well. Then, 100 µL of various bacterial strains with 1.5 10^6^ CFU/mL were added to all but the negative control well. The microplates were incubated for 24, 48, and 72 h at 37 °C [36, 37].

### Investigation of biofilm inhibition

To quantitatively assess biofilm formation in the presence and absence of nano and free medicines, the Crystal violet staining method was used. In summary, fresh cultures of various strains were diluted 1:100 and added to the TSB culture medium that had been enhanced with 1% glucose. Then, various amounts of PLGA-VAN, PLGA-VAN-LYS, and Free vancomycin were applied to each microplate well (the final concentrations in each well were 50, 30, 20, and 5 µg/mL). For 24, 48, and 72 h, the microplate was incubated at 37˚C. Each well's medium was then gradually withdrawn, and PBS was used to wash it three times. The biofilm in each well was stabilized with methanol. Next, crystal violet (2%) was used to stain the wells. The wells were gently cleansed with water after 15 min. The second stage was obtaining an adherent cell suspension in 95% ethyl alcohol. At 600 nm, optical density was identified after 30 min [38].

### Statistical analysis

The Analysis of Variance (ANOVA) test was used to examine the differences between the treatments. The Dunnett test was another statistical analysis used to compare groups. Statistical significance was determined to be *P* < 0.05, and the confidence interval was established at 95%.

## Results

### Characteristics of the NPs

In the optimum formulation of PLGA-VAN-LYS (F4), the mean diameter, PDI, and zeta potential of the NPs had been 320.5 ± 35 nm, 0.270 ± 0.012, and -19.5 ± 1.3 mV, respectively (Table 1).

**Table 1:** Substances and technological parameters of diverse formulation

Formulation	VAN (mg)	LYS (mg)	PLGA (mg)	DCM^a^ (ml)	Chl^b^ (ml)	PVA (ml)	Size (nm)	PDI	ZP (mV)	EE (%)	DL (%)	LCE^c^ (%)
F1	6	-	30	20	-	20 (1%)	674.4 ± 23	0.386 ± 0.024	-17.2 ± 2.3	91.35 ± 6.2	14.25 ± 2.5	**-**
F2	6	-	30	-	15	20 (1%)	494.5 ± 27	0.292 ± 0.026	-18.7 ± 1.8	92.46 ± 3.4	15.27 ± 2.7	**-**
F3	12	-	60	-	15	2 (2%)	388.6 ± 38	0.279 ± 0.015	-21.2 ± 1.4	94.35 ± 2.5	16.23 ± 1.3	**-**
**F4**	**24**	**3**	**120**	**-**	**15**	**20 (2%)**	**320.5 ± 35**	**0.270 ± 0.012**	**-19.5 ± 1.3**	**94.62 ± 2.6**	**16.75 ± 2.5**	**37**

^a^Dichloromethane

^b^Chloroform

^c^Lysostaphin conjugation efficiency

### Morphology

**Fig. 1:**
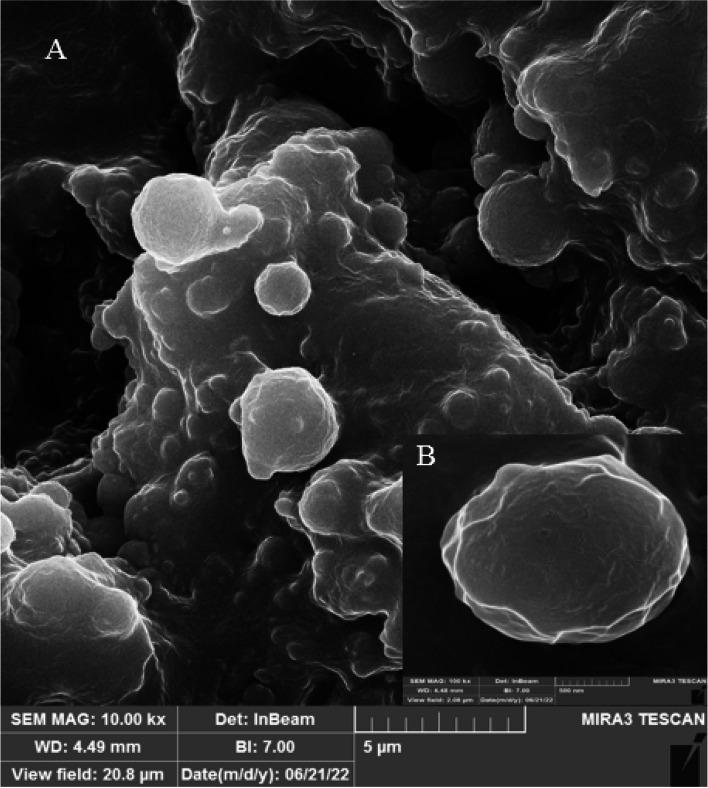
**A**) Field emission scanning electronic microscopy of PLGA-VAN, **B**) the magnified part of the PLGA-Van

The results of the FE-SEM investigation of the PLGA-VAN morphology are displayed in Fig. 1. The majority of the particles appeared to be spherical and had a smooth surface with some homogenous dispersion, as seen by the photograph. The majority of the NPs were about the same size, and PDI (0.270 ± 0.012) supports this finding.

### Drug loading and encapsulation efficiency

Vancomycin was loaded into PLGA in varying amounts, ranging from 14.25% to 16.75%, and it was encapsulated to varying degrees (91.35% to 94.62%). The loaded and encapsulated vancomycin levels for the ideal PLGA-VAN-LYS (F4) formulation were 16.75% ± 2.5 and 94.62% ± 2.6, respectively (Table 1).

### Stability of NPs

At 0, 2, 4, 6, 8 and 12 months after manufacture, nanoparticles' particle size, PDI, and zeta potential were assessed (Table 2). According to the results, there was a very tiny difference in size between the nanoparticles up until the sixth month after manufacture. After 12 months, these sizes increased from 320.5 ± 35 nm to 495.5 ± 34 nm, this increase was statistically significant (*P* < 0.05). Changes in PDI and zeta potential were not significant.

**Table 2:** Technological characteristics of the PLGA-VAN formulation: average diameter, PDI and zeta potential throughout stability study, (means ± SD, *n* = 3)

Formulation	Technological parameters	Time (months)					
		0	2	4	6	8	12
**PLGA-VAN-LYS (F4)**	Size (nm)	320.5 ± 35	322.5 ± 32	326.3 ± 25	332.8 ± 34	420.4 ± 42	495.5 ± 34
	PDI	0.270 ± 0.012	0.270 ± 0.017	0.274 ± 0.010	0.278 ± 0.014	0.285 ± 0.012	0.290 ± 0.011
	ZP^a^ (mV)	-19.5 ± 1.3	-19.2 ± 3.8	-18.9 ± 2.1	-18.5 ± 2.3	-17.9 ± 3.4	-17.5 ± 3.8

^a^Zeta potential

### Drug release

**Fig. 2:**
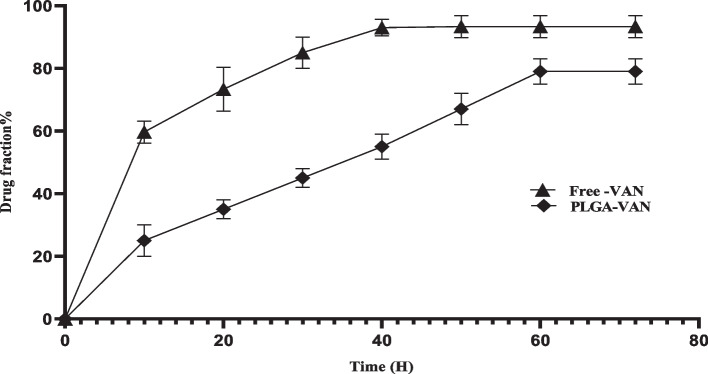
In vitro release profiles of vancomycin from the PLGA-VAN-LYS (F4) formulation in pH = 7.4 phosphate buffer. Free vancomycin became used as control

Figure 2 depicts the results of the drug release test, which was carried out in 72 h (In vitro, pH: 7.4, PBS buffer). The results showed that more than 72 h were needed to release about 80% of the drug from the PLGA matrix. In contrast, 35% of the drug was released from the PLGA matrix in the first 20 h, whereas 80% of the free drug was released after 20 h.

### FTIR analysis

**Fig. 3:**
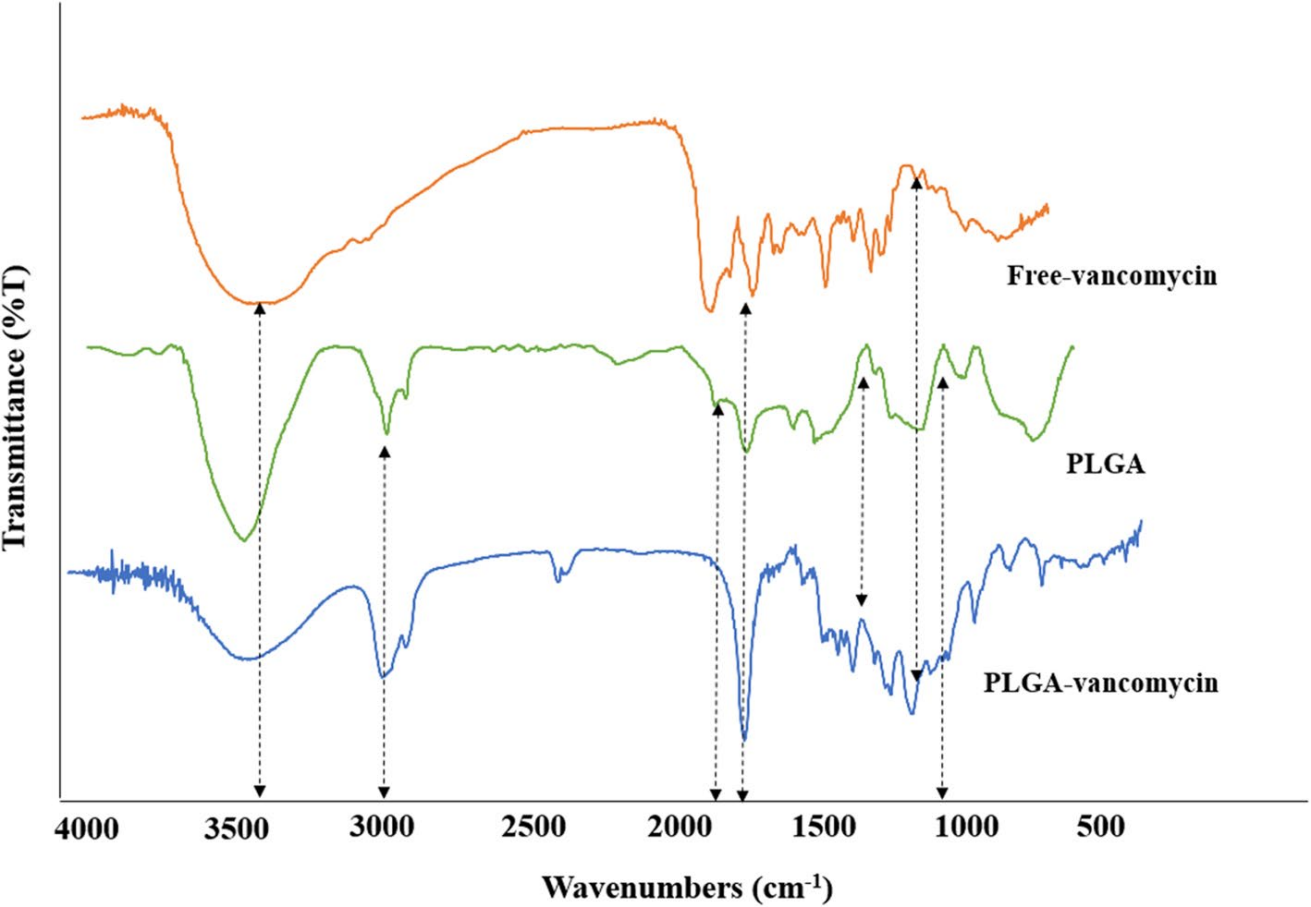
FTIR spectra of free vancomycin (Free-VAN), PLGA and PLGA-VAN

FTIR spectra of vancomycin, PLGA, and PLGA-VAN are shown in Fig. 3. As seen in Fig. 3, Vancomycin showed peaks at 3,440 cm^−1^ and 1,635 cm^−1^, which corresponded to the -OH stretching and the C = O vibration, respectively. PLGA showed peaks at 3450 cm^−1^, 3010 cm^−1^, 1762.6 cm^−1^ and 1186 cm^−1^, which corresponded to the –OH end group, C-H stretches, C = O stretch and C-O stretch respectively. The FTIR spectrum of PLGA-VAN showed all the characteristic peaks of Van and PLGA, confirming that Vancomycin was successfully encapsulated in PLGA-VAN.

### DSC analysis

**Fig. 4:**
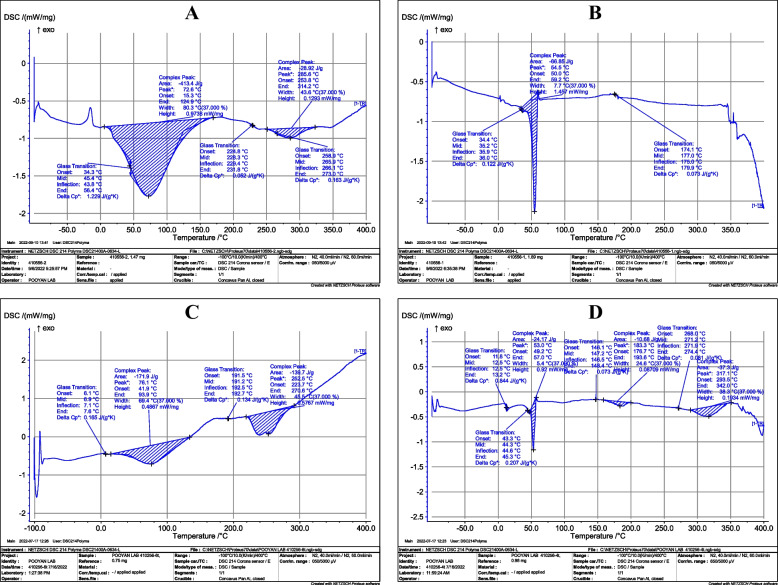
DSC thermograms of **A**) Free-VAN, **B**) PLGA, **C**) PLGA + Free-VAN (physical mixture), **D**) PLGA-VAN

DSC thermograms of vancomycin, PLGA, physical mixture, and PLGA-VAN nanoparticles are received in order to study the recrystallization and melting behavior of PLGA nanoparticles (Fig. 4). The DSC thermogram of PLGA shows that melting occurs at 54.5 °C. The physical mixture's and the PLGA-VAN nanoparticles' melting points were quite similar to those of PLGA. In the physical mixing with the vancomycin, a little modification in the PLGA's melting process was found. A distinct endothermic peak was found in the DSC thermograms of vancomycin at 258.9 °C. The melting point for the physical mixture and PLGA-VAN is low, according to data on this point for other compounds. It's interesting to note that the endothermic peak positions, physical mixture, and PLGA-VAN of vancomycin did not significantly change. The lack of a distinct melting peak on the PLGA-VAN thermogram suggests that the vancomycin molecules are stabilized inside the PLGA matrix or that there is no free vancomycin crystal left in the PLGA-VAN.

### MTT assay

**Fig. 5:**
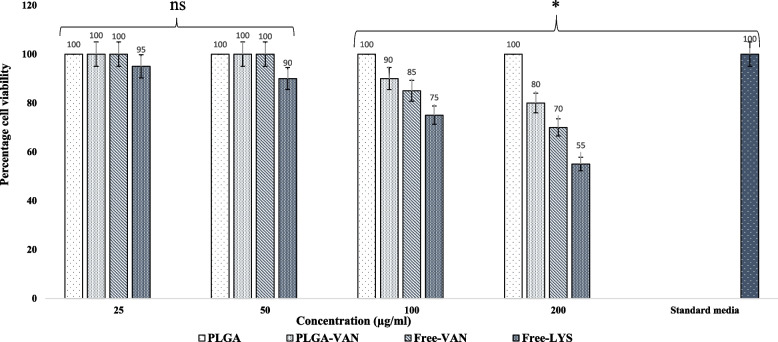
The effects of PLGA-VAN, Lysostaphin and Free-VAN on L929 cells, NS, non-significant; *p <0.05

Figure 5 illustrates the effects of various concentration of PLGA-VAN, vancomycin, free PLGA (blank), and lysostaphin on murine L929 fibroblasts. At 37 °C and with 5% CO_2_, cells were incubated with different doses of free vancomycin, PLGA-VAN, free PLGA, and lysostaphin. Additionally, the exact same cells that were used in the positive controls (without treatment) were incubated in the culture medium. With the exception of lysostaphin, none of the formulations were hazardous at 50 µg/ml. At 200 µg/ml, PLGA-VAN was less hazardous than free vancomycin.

### Agar well diffusion and Minimum inhibitory concentration (MIC)

The well diffusion and MIC test results at 24, 48, and 72 h are shown in Tables 3 and 4. In both methods, at 24 and 48 h, free vancomycin had a better effect on all four strains than PLGA-VAN. These outcomes were expected since, in both procedures, the bacteria came into touch with the free drug whereas only a little amount of the drug was released from PLGA-VAN. Additionally, during 72 h of incubation, the drug was gradually released from PLGA-VAN, increasing the inhibitory zone of PLGA-VAN. Additionally, after 72 h, the MIC of PLGA-VAN was closer to the free vancomycin. Nanoparticles conjugated with lysostaphin did not have any inhibitory effect on *Staphylococcus aureus* strains at 24, 48 and 72 h of incubation.

**Table 3:** Results of MIC

MIC value (µg/ml) 150–100-50–25-15–5-0.5 (µg/ml)			
**Time (h)**	Bacterial strains	PLGA-VAN	Free-VAN
**24h**	MSSA	25	0.5
	MRSA	100	0.5
	VISA	50	5
	Clinical strain	50	0.5
**48h**	MSSA	15	0.5
	MRSA	50	0.5
	VISA	15	5
	Clinical strain	50	0.5
**72h**	MSSA	5	0.5
	MRSA	25	0.5
	VISA	15	5
	Clinical strain	15	0.5

**Table 4:** Results of Agar well diffusion

Zone of inhibition (mm) 50–25-5 (µg/ml)							
**Time (h)**	Bacterial strains	PLGA-VAN			Free-VAN		
**24h**	MSSA	30	20	15	55	45	40
	MRSA	20	10	5	50	35	30
	VISA	20	15	5	35	30	25
	Clinical strain	30	20	10	45	40	30
**48h**	MSSA	35	25	20	55	45	40
	MRSA	25	15	15	50	35	30
	VISA	25	20	5	35	30	25
	Clinical strain	30	25	15	45	40	30
**72h**	MSSA	40	30	30	55	45	40
	MRSA	35	25	20	50	35	30
	VISA	30	25	15	35	30	25
	Clinical strain	35	30	15	45	40	30

### Biofilm analysis

**Fig. 6:**
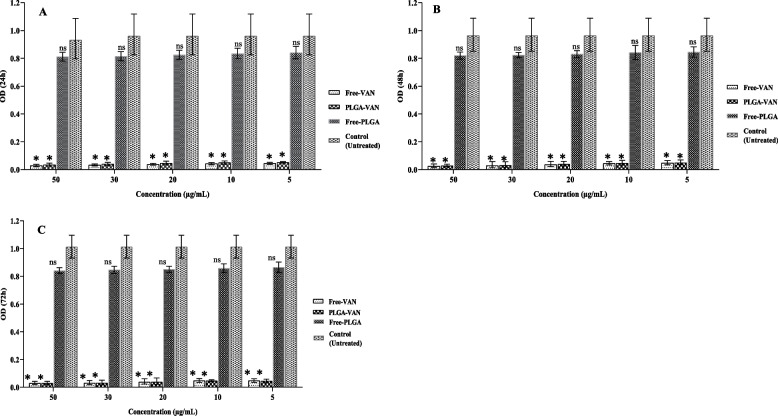
Graphical depiction of biofilm inhibition (OD values) of methicillin-sensitive Staphylococcus by different formulations, in **A** 24, **B** 48 and **C** 72 h. Comparisons were performed between free VAN, PLGA-VAN, free-PLGA and control (treatment): NS, non-significant; *p < 0.05

**Fig. 7:**
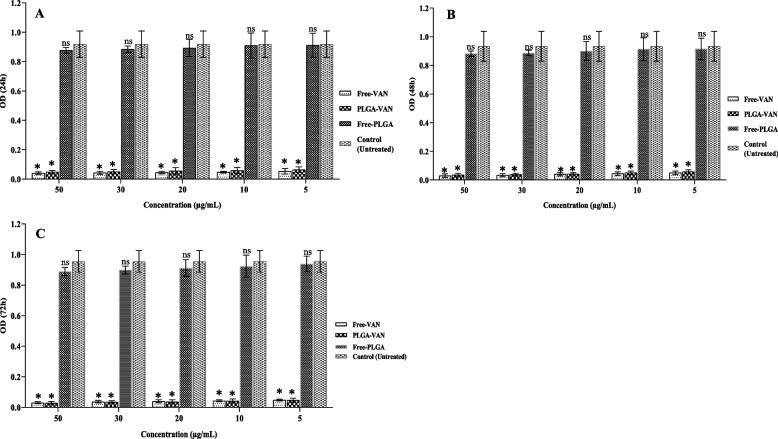
Graphical depiction of biofilm inhibition (OD values) ofmethicillin-resistance Staphylococcus by different formulations, in **A** 24, **B** 48 and **C** 72 h. Comparisons were performed between free VAN, PLGA-VAN, free-PLGA and control (treatment): NS, non-significant; *p < 0.05

**Fig. 8:**
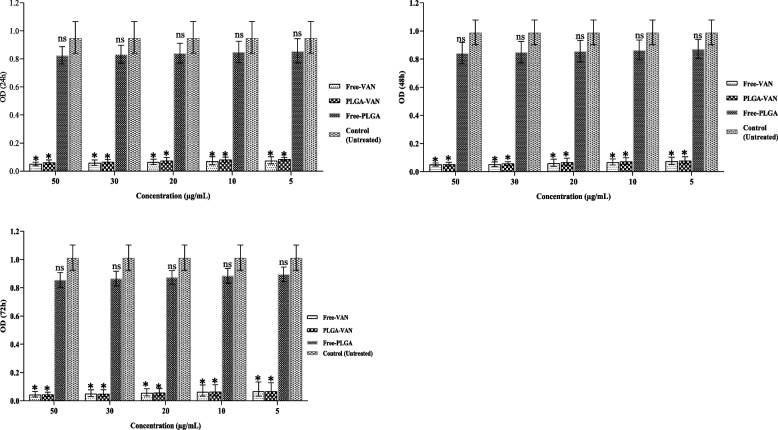
Graphical depiction of biofilm inhibition (OD values) of vancomycin intermediate Staphylococcus by different formulations, in A) 24, B) 48 and C) 72 h. Comparisons were performed between free VAN, PLGA-VAN, free-PLGA and control (treatment): NS, non-significant; *p < 0.05

**Fig. 9:**
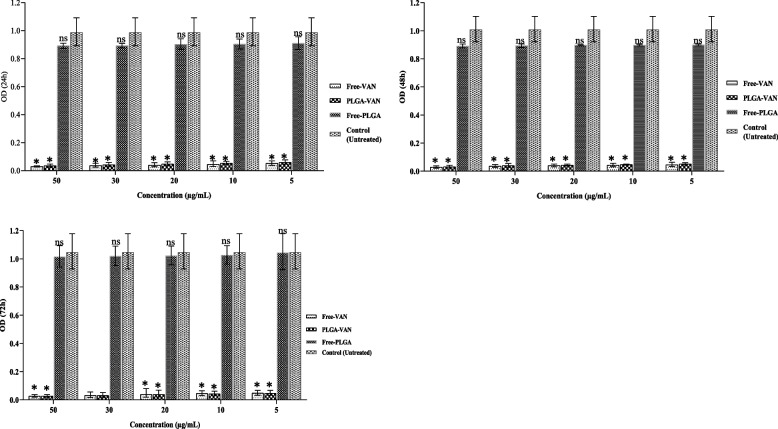
Graphical depiction of biofilm inhibition (OD values) of clinical strain Staphylococcus by different formulations, in A) 24, B) 48 and C) 72 h. Comparisons were performed between free VAN, PLGA-VAN, free-PLGA and control (treatment): NS, non-significant; *p < 0.05

The results associated with the effects of PLGA-VAN and free vancomycin on the biofilm inhibition of MSSA, MRSA, VISA, and clinical strains at 24, 48, and 72 h are shown in Figs. 6, 7, 8 and 9 respectively. PLGA-VAN and free vancomycin were effective in biofilm inhibition at 24, 48 and 72 h of incubation. In contrast, both the strains exposed to free PLGA and the positive control strains (not treatment, formed a strong biofilm. At 24 and 48 h, free vancomycin more inhibited biofilm formation by all four strains compared to PLGA-VAN. After 72 h, the effects of PLGA-VAN increased due to the gradual release of vancomycin, and the results of PLGA-VAN at 72 h were close to the results of free vancomycin, and in some cases, the effect of PLGA-VAN was even more. Nanoparticles conjugated with lysostaphin had no effect on inhibiting biofilm formation at 24, 48 and 72 h of incubation and the strains exposed to them formed strong biofilm.

## Discussion

There are various methods for drug loading in PLGA, which are selected based on the type of drug and study’s objectives. Nanoprecipitation and emulsion solvent evaporation are two common methods for drug loading in PLGA [39]. In our study, we used the double emulsion evaporation method due to the hydrophilic nature of vancomycin. The average size of the synthesized nanoparticles in the optimum formulation was 320.5 ± 35 nm, which was suitable for our final goal of topical application. Our study showed that various factors affect the size of the nanoparticles. Initially, we used dichloromethane as the solvent for PLGA, which resulted in larger nanoparticle size. Finally, we used chloroform in the optimum method, which dissolved PLGA well and reduced the particle size. PVA is a surfactant and emulsifier that stabilizes two phases in an emulsion and affects the size of nanoparticles [40]. In our investigation, using 2% PVA reduced the average size of nanoparticles compared to using 1% PVA. This was in line with the research conducted by Posadowska et al. [40]. Additionally, the length of the sonication has an impact on particle size. According to the results of Rui et al., [30] the particle size in our investigation decreased as the sonication period increased. PDI indicates the degree of heterogeneity of particle size in a colloidal system [41]. In our study, the PDI size in the optimum formulation was 0.270 ± 0.012, indicating that the particles were homogeneous in terms of size, which was consistent with the electron microscopy images. Moreover, in our study, the PDI decreased with an increase in sonication time, which was consistent with the findings of Ruiz et al. [41]. In our study, the zeta potential was – 19.5 ± 1.3 mV. The negative zeta potential increases the stability of particles as repulsive forces prevent particle aggregation over time [42]. Therefore, in our study, the synthesized nanoparticles were stable. One of the factors that affect the zeta potential is the amount of PVA used. In our study, the zeta potential was more negative when 2% PVA was used compared to when 1% PVA was used, that was consistent with the study conducted by Sahoo et al. [43]. In our study, the amount of vancomycin loading and encapsulation in the optimum formulation was 16.75 ± 2.5% and 94.62 ± 2.6%, respectively. As we increased the amount of drug used, the drug loading in PLGA increased, which was consistent with the study conducted by Snejdrova et al. [44]. Our findings showed that with an increase in the duration of ultrasound probe usage, the drug loading and encapsulation in PLGA decreased. Therefore, the duration of ultrasound probe usage in the optimum method was two minutes. These findings were consistent with the study by Ito et al. [45]. Consequently, as the homogenization time was increased, the drug loading in PLGA reduced. The stability of the ideal formulation was examined in our investigation during a 12-month period. The nanodrug's particle size rose from 320.5 ± 35 nm to 495.5 ± 34 nm after 12 months of synthesis, which may have been caused by the agglomeration of nanoparticles. However, the zeta potential and PDI showed no discernible difference, which was consistent with the research of Hosseini et al. [46]. One of the benefits of drug loading in PLGA is the controlled drug release at the desired site by the degradation of the PLGA polymer matrix. As a result, drug stability at the site of action increases. In this study, the drug release duration from PLGA using a dialysis bag was investigated. Our findings showed that, in the initial 10 h, 25% of vancomycin was released from PLGA-VAN, while during the same period, 60% of Free-VAN was released. The drug release study demonstrated that it takes approximately 72 h for 80% of vancomycin to be released from PLGA. The drug release duration from different nanoparticles in various studies varies due to the use of different methods and materials. Generally, in most studies, the drug release duration from nanoparticles is longer than that of free drugs. The slow drug release from PLGA into lactic acid and glycolic acid monomers. In the study by Topal et al. [29] vancomycin was completely released into the release medium in the first 6 h, while the release of vancomycin during the same period from different PLGA formulations was between 25 to 40%. Since the ultimate goal of using nanodrugs is to treat disease in humans, the assessment of nanodrug toxicity is of paramount importance. In the present study, the toxicity of synthesized nanodrugs was investigated on L929 fibroblast cell line. The results showed that the cell viability percentage in the concentration of 200 µg/mL PLGA-VAN and Free-VAN was 80% and 70%, respectively. Therefore, with vancomycin encapsulation in PLGA, cellular toxicity was reduced. In the study by Rui et al. [30] the cell viability percentage at the concentration of 100 µg/mL PLGA-VAN was higher than that of Free-VAN. In our study, after investigating the characteristics of synthesized nanodrugs using agar well diffusion, MIC, and evaluating their ability to inhibit biofilm formation, their antimicrobial activity was evaluated. The results of agar well diffusion and MIC showed that in the initial 24 h, the antimicrobial activity of the free drug on *S. aureus* strains was higher than that of synthesized nanodrugs. However, after 72 h of incubation, the antimicrobial activity of synthesized nanodrugs increased due to the release of high mounts of the drug and became close to that of the free drug. This is because the drug release duration in our study was 72 h. In the study by Le et al. [47] the MIC and MBC of Free-Lev were lower than that of PLGA-Lev on *S. aureus* strains, which they attributed to the incomplete drug release from PLGA. As mentioned, it took approximately 72 h for the drug to be released from PLGA. The slow and controlled release of the drug from nanoparticles has various benefits on the biofilm inhibition, including increased drug bioavailability and local drug concentration. This leads to enhanced interaction between the drug and bacterial cells within the biofilm and improves treatment efficacy. Additionally, the drug’s half-life is increased at the site of the biofilm. The evaluation of biofilm inhibition in our study revealed that at 24, 48, and 72 h, PLGA-VAN and Free-VAN significantly differed from the control group (no treatment), and biofilm formation was decreased in all three instances. However, pure PLGA did not have a significant difference with the control group and had no antibiofilm activity in all three time. After 72 h, the results of PLGA-VAN were better than Free-VAN at some concentrations, although this difference was not significant. The results of the study by Ustun et al. [34] showed that PLGA nanoparticles loaded with nisin were able to effectively inhibit *S. aureus* biofilm formation, so that PLGA loaded with nisin inhibited 72% of the biofilm formation, while free nisin inhibited 28% of the biofilm formation. In our study, lysostaphin was conjugated with PLGA by using chemical compounds EDC and NHS, which created a bond between the carboxyl end of PLGA and the amine end of lysostaphin. The percentage of lysostaphin conjugation was measured to be 37% in the optimum formulation using the Bradford kit. In the study by Moura et al. [31] the percentage of conjugation of anti-CD46 on PLGA was between 31 to 36% in different formulations. In the present study, lysostaphin-conjugated nanodrugs, unlike PLGA-VAN, had no antimicrobial and anti-biofilm activity and had no inhibitory effect in agar well diffusion, MIC, and biofilm formation inhibition tests. The reason for this could be the long conjugation process of lysostaphin, which resulted in drug release during the process, and the drug was removed by centrifugation.

## Conclusion

The double emulsion evaporation method was successfully synthesized, which can be a potential candidate for treating *S. aureus* infections and inhibiting biofilm formation. The antibacterial activity of free vancomycin compared to PLGA-VAN was higher in MIC and well diffusion methods at 24 and 48 h because the release time of vancomycin from PLGA-VAN was approximately 72 h. Therefore, the effectiveness of PLGA-VAN in the well diffusion and MIC methods was 72 h. Also, compared to PLGA-VAN, free vancomycin had a more significant effect on inhibiting the biofilm formation of *S. aureus* strains. While their results were almost the same and even slightly better in some cases after 72 h. Therefore, the results of our study showed that the effectiveness of optimum formulation of PLGA-VAN is promising after 72 h and use of this technology can have favorable effects to reduce staphylococcal infections and a promising tool to deal with hospital infections caused by this bacterium.

## Data Availability

The datasets used and/or analyzed during the current study are available from the corresponding author on reasonable request.
